# Literary and Journalistic Notes

**Published:** 1905-03

**Authors:** 


### LITERARY AND JOURNALISTIC NOTES.

THE *Iowa Medical Journal,* in its January issue, published a directory of Iowa physicians, arranged by counties. This is followed by an alphabetical list, the whole constituting an enormous labor and no inconsiderable cost. The enterprise of this sterling journal is to be commended. It is generally speaking, a thankless task to prepare such lists, but we hope the journal's work will be appreciated by its numerous readers, not only within the state but outside of it.

THE *Georgia Practician* is the name title of a new medical publication, edited by Martin Cooley, M.D., and published at Savannah. It begins with a lesson in philology and serves notice on the exchange bureau of diagnosis-and-treatment that it will have none of it. We wish the *Practician* success.

"KING'S Medical Prescriptions," is the title of a book containing selected formulas from prominent physicians, published by the Practice Science Company, 108 Fulton street, New York. Superfine paper; large type; octavo, 346 pages. Second edition; cloth, $1.00, or paper covers, 50 cents, postpaid.

